# Graph-KIR: graph-based KIR copy number estimation and allele calling using short-read sequencing data

**DOI:** 10.1093/bioinformatics/btag521

**Published:** 2026-07-21

**Authors:** Hong-Ye Lin, Ting-Jian Wang, Ting-Yu Chang, Hui-Wen Chuang, Tsung-Kai Hung, Ching-Jim Lin, Jacob Shujui Hsu, Chia-Lang Hsu, Ya-Chien Yang, Pei-Lung Chen, Chien-Yu Chen

**Affiliations:** Department of Biomechatronics Engineering, National Taiwan University, Taipei 10617, Taiwan; Department of Biomechatronics Engineering, National Taiwan University, Taipei 10617, Taiwan; Graduate Institute of Medical Genomics and Proteomics, National Taiwan University, Taipei 10055, Taiwan; Graduate Institute of Medical Genomics and Proteomics, National Taiwan University, Taipei 10055, Taiwan; Graduate Institute of Medical Genomics and Proteomics, National Taiwan University, Taipei 10055, Taiwan; Graduate Institute of Biomedical Electronics and Bioinformatics, National Taiwan University, Taipei 10617, Taiwan; Graduate Institute of Medical Genomics and Proteomics, National Taiwan University, Taipei 10055, Taiwan; Department of Medical Research, National Taiwan University Hospital, Taipei 10002, Taiwan; Department of Clinical Laboratory Sciences and Medical Biotechnology, National Taiwan University College of Medicine, Taipei 10051, Taiwan; Graduate Institute of Medical Genomics and Proteomics, National Taiwan University, Taipei 10055, Taiwan; Department of Biomechatronics Engineering, National Taiwan University, Taipei 10617, Taiwan

## Abstract

**Motivation:**

The Killer-cell Immunoglobulin-like Receptor (KIR) is a highly polymorphic region in the human genome, associated with autoimmune diseases and organ transplantation. The sequences of KIR genes are highly similar among star alleles as well as in between individual genes, with the copy number of each KIR gene typically ranging from 0 to 4. In this study, we introduce Graph-KIR, a tool designed to estimate gene copy numbers and predict full-resolution (7-digit, encompassing both coding and non-coding sequence variations) from a whole genome sequencing (WGS) sample.

**Results:**

Graph-KIR is capable of independently typing KIR alleles per sample with no reliance on the distribution of any framework gene in a cohort. In a set of 100 simulated samples, Graph-KIR demonstrated 99.2% accuracy in copy number estimation and high F1-score of allele typing: 91.79% at 7-digit resolution, 97.37% at 5-digit resolution, and 97.11% at 3-digit resolution. Graph-KIR outperforms existing tools such as Geny (96.39% F1-score), PING’s WGS version (92.77% F1-score), and T1K (90.44% F1-score) at 5-digit resolution. By analyzing the results on 44 HPRC samples, Graph-KIR achieves better F1-score than Geny and PING at 7-digit resolution. The release of Graph-KIR adds another valuable tool to assist users in accurately estimating copy numbers and calling alleles of KIR genes from WGS samples.

**Availability and implementation:**

The Graph-KIR and paper-related pipeline codes are available at https://github.com/linnil1/KIR_graph.

## 1 Introduction

The Killer-cell Immunoglobulin-like Receptor (KIR) family plays a crucial role in the operation of natural killer (NK) cells, controlling the cell-killing mechanism through its binding with Major Histocompatibility Complex (MHC) class I molecules on the cell surface (Pende *et al.* 2019). Such interactions are gene-specific to both the Human Leukocyte Antigen (HLA) and KIR. Some studies (Carr *et al.* 2005, Yawata *et al.* 2006) have shown that certain ligations are gene content-specific, meaning that they depend not only on the types of genes present but also on the star alleles. Consequently, accurately calling the star alleles of all KIR genes in an individual is of significant importance for future research and clinical applications.

The KIR gene family, ranging from 70 to 270 kilobase pairs (kbp) in length, is located on chromosome 19 (19q13.4). This family encompasses 15 genes (*KIR2DL1/L2/L3/L4*, *KIR2DL5A*, *KIR2DL5B*, *KIR2DS1/S2/S3/S4/S5*, *KIR3DL1/S1*, *KIR3DL2/L3*) and two pseudogenes (*KIR2DP1* and *KIR3DP1*). All KIR genes, spanning approximately 5–13 kbp each, exhibit a notable similarity in both their DNA sequence structures and contents. Aside from the similarity across genes, the complex internal similarity within each gene is analogous to another immune gene family, HLA. The star allele of a KIR gene, for instance, KIR2DL1*0010101, represents a set of single nucleotide polymorphisms (SNPs) across the entire gene. The classification of star alleles is divided into three distinct resolution levels: identical protein sequences, identical DNA exon regions, and identical DNA sequences, corresponding to 3-digit, 5-digit, and 7-digit, respectively, according to the KIR nomenclature definition (Marsh *et al.* 2003). Each KIR gene typically has 0–4 star alleles in an individual, based on the KIR haplotype-level polymorphism, the value we refer to as the copy number (CN). All of these concepts are illustrated in Fig. S1, available as supplementary data at *Bioinformatics* online.

KIR typing is usually divided into two tasks: Copy Number Estimation (addressing the gene level and copy number level) and Allele Typing (dealing with the star-allele (SNP) level). These two steps are crucial for KIR typing and are the common components of existing KIR tools. To the best of our knowledge, there are at least six tools available for identifying KIR genes from high-throughput genotyping or sequencing data: KIR*IMP (Vukcevic *et al.* 2015), KPI (Roe and Kuang 2020), PING (Norman *et al.* 2016, Marin *et al.* 2021, Marin and Hollenbach 2023), the pipeline (denoted as SakaueKIR in this study) developed by Sakaue *et al.* (Sakaue *et al.* 2022), T1K (Song *et al.* 2023), and Geny (Zhou *et al.* 2024a). However, KIR*IMP does not support short-read sequencing data and KPI lacks the allele typing step. In a strict sense, only three tools are capable of accepting read files in FASTQ format as input to generate KIR allele typing results.

For the task of copy number estimation, one typical way is to determine the copy numbers of genes based on their corresponding read depths. The CN of a gene is highly related to its read depth in the short-read sequencing data. For example, when there are three alleles belonging to *KIR2DL1* (CN = 3), all the reads originating from those three alleles will be mapped to *KIR2DL1*, resulting in the read depth of *KIR2DL1* being about 1.5 times higher than the normal read depth (i.e. diploid, CN = 2). By gathering the read depths of genes, those values can be clustered into copy number groups by specific thresholds. PING carefully selects numerous probes to determine the read depths of each gene by counting the reads matching the probes followed by normalization based on the framework gene *KIR3DL3* (assuming *KIR3DL3* always has two copies). When using PING, the thresholds of each CN group are manually determined. SakaueKIR uses average read depths directly calculated from pre-selected regions and clusters them by Kernel Density Estimation (KDE). Both PING and SakaueKIR require large sample sizes in a cohort for clustering. T1K estimates allele abundance by EM algorithm, but it limits to at most two alleles per gene, which does not meet the KIR complexity because a KIR gene may has more than two copies.

From an allele typing perspective, star alleles should be reported along with the estimated copy number of each gene. PING iteratively maps reads to the star allele candidates and gets the final 5-digit alleles. SakaueKIR uses a GATK-like pipeline and determines alleles by finding a set of star alleles that fits the partially phased SNPs calculated by HaplotypeCaller (Poplin *et al.* 2017). T1K tries to maximize the likelihood by choosing one or two alleles according to their abundance. Additionally, Geny specifically prioritizes functional allele typing, with a primary focus on 3-digit resolution. Despite these advancements, a gap remains as none of the current methods provide comprehensive, full-resolution typing of KIR star alleles.

For clinical practices, it is better for a tool being able to update its KIR allele sequence database to keep up with the official KIR database Immuno Polymorphism Database-KIR (Robinson *et al.* 2012) (IPD-KIR), which collects the names of star alleles and their corresponding sequences reported so far. Recent published tools like T1K can update its database, while SakaueKIR uses IPD-KIR 2.7.0 and PING uses IPD-KIR v2.7.1 stated in the published papers.

To conquer the difficulty oriented to the high similarity between KIR genes and KIR star alleles mentioned above, inspired by HISAT-genotype (Kim *et al.* 2019), we propose a novel pipeline, Graph-KIR. Similar to the solution provided by HISAT-genotype for typing HLA star alleles, Graph-KIR utilizes HISAT2, a graph read mapper, to map short reads to custom-built indexes. The highly accurate graph mapping enables Graph-KIR to estimate copy number per sample independently, thanks to the higher linearity between copy number and read depth in the graph alignment. The task of allele typing relies on the variants called from the graph, and a set of star alleles is called by maximum likelihood estimation. Furthermore, any version of IPD-KIR database can be converted to graph indexes using the tool provided by HISAT2 along with the custom codes together released with Graph-KIR.

In this study, Graph-KIR uses all the sequences in IPD-KIR v2.10.0, including 879 full-length sequences and 652 partial sequences (exon-only sequences). In total, 1110 distinct star alleles are used in building the graph index. Each KIR gene has at least 30 alleles. Regarding performance, Graph-KIR was tested on both simulated and real WGS samples. Several tools have been developed to annotate real samples using high-quality phased assemblies (Ford *et al.* 2024, Hung *et al.* 2024, Zhou *et al.* 2024b). The annotation of real samples in Graph-KIR was provided by one of these studies (Hung *et al.* 2024).

## 2 Materials and methods

### 2.1 Pipeline overview

**Figure 1:**
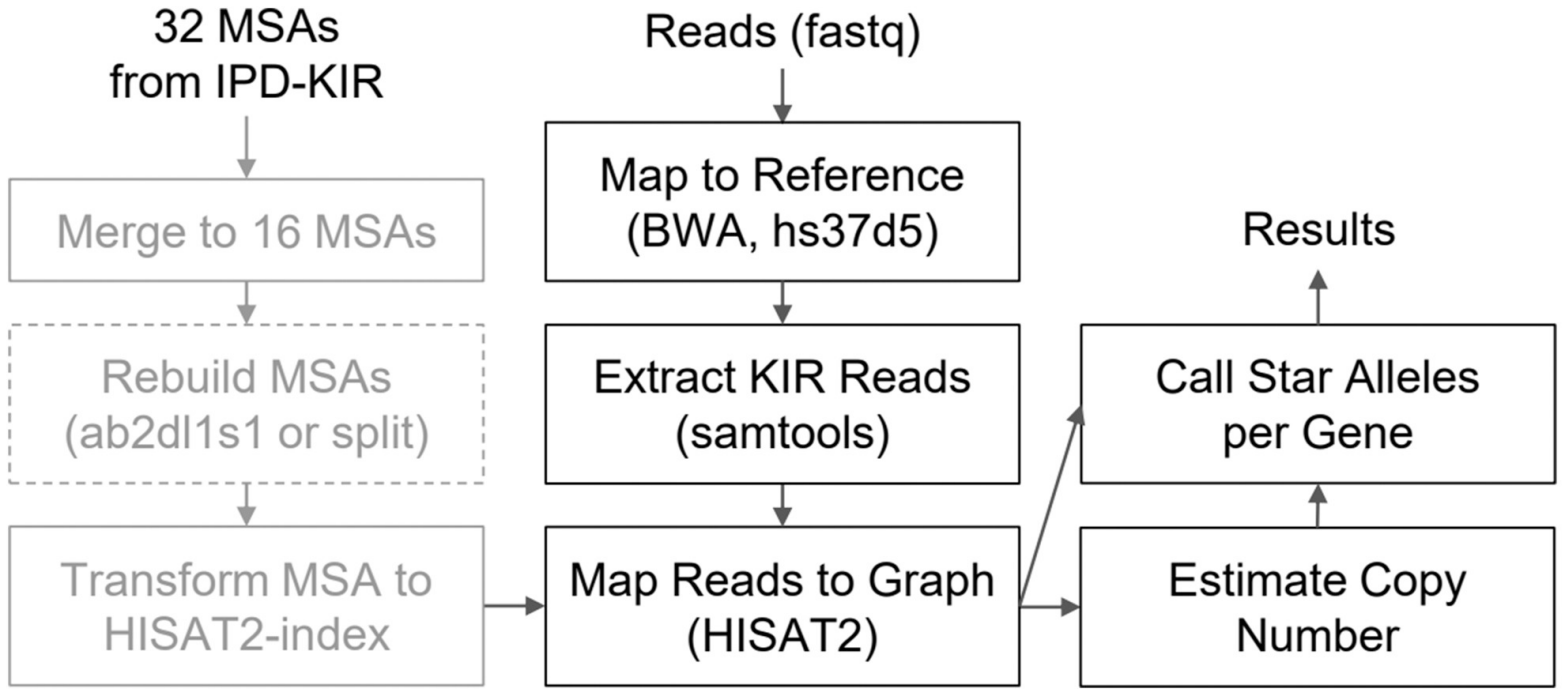
Overview of the Graph-KIR pipeline. The preprocessing steps that only need to be executed once are highlighted in grey, and the optional step is represented by a dashed box.

Graph-KIR contains five main steps: (i) building graph index from MSA; (ii) read mapping to the human genome and extracting KIR-related reads (optional); (iii) graph read mapping and read filtering; (iv) copy number estimation; and (v) allele typing, as illustrated in Fig. 1.

The graph index is built from the MSAs of all alleles for the 17 KIR genes available from IPD-KIR. These MSAs are manipulated by merging similar genes (*KIR2DL1* and *KIR2DS1*), left aligning, and adding pseudo introns to exon-only sequences, making the MSAs more suitable for graph index building and read mapping. Once the index is built, there is no need to rebuild it unless the database version of IPD-KIR changes.

The whole genome pair-end reads are first mapped to the human genome hg19. The reads mapped at KIR-related regions are extracted. If the input reads are already filtered for KIR regions, this step can be skipped.

Next, the reads are mapped to the graph index by HISAT2. Here, Graph-KIR discards multi-mapped reads and reads with low alignment scores because such reads may result in overestimation of read depth and cause false positives in variant calling.

The key step in copy number estimation of Graph-KIR is distribution fitting. Multiple distributions, each representing one of the copy numbers (0–6), are fit to read depths calculated from all the bases of each gene. The distributions have constraints about the linearity between copy number and the estimated read depth. This feature enables estimating copy numbers with only one sample while maintaining its robustness and high accuracy.

After copy number estimation, Graph-KIR calls a set of alleles for each gene with its size equal to the copy number determined previously. This allele typing step includes calling variants of reads from the graph, comparing the variants with the star alleles, assigning each read to an allele in the set, and iteratively finding the best-fitting allele set.

### 2.2 MSA generation and index preparation

The MSAs are reconstructed from .msf and .dat files downloaded from IPD-KIR database. Each gene contains both exon-only and full-length MSA. In total, there are 32 MSAs from 17 genes, where *KIR2DL5A* and *KIR2DL5B* are already merged.

The MSAs in Graph-KIR are merged version that preserves both introns and exons information, combining the exon alignment from the exon-only MSA and the intron alignment from the full-length MSA. For exon-only alleles, i.e. sequences missing intronic regions, the introns are filled by consensus calculated from similar alleles in the same gene.

The recommended graph index for the Graph-KIR method is “ab2dl1s1,” constructed from 15 MSAs. In this index, *KIR2DL1* and *KIR2DS1* are merged, as are *KIR2DL5A* and *KIR2DL5*. For comparison, two alternative indices are used: “ab” (16 MSAs, with *KIR2DL5A* and *KIR2DL5B* merged) and “split” (17 MSAs, with no merging).

The last part of MSA processing is left-aligning, inspired by the VCF normalization (Tan *et al.* 2015). Here, all deletions in the MSA will be left-aligned.

### 2.3 Read extraction

Before entering the core Graph-KIR algorithm, the whole genome sequencing (WGS) reads are first mapped to the hg19 reference genome. Graph-KIR uses the version hs37d5 of hg19 obtained from 1000 Genomes Project (1000 Genomes Project Consortium 2015). Read mapping is run by BWA-MEM (Li 2013) with parameters -M -K 100000000-a. Then, the reads mapped to KIR regions are extracted via Samtools (Danecek *et al.* 2021) but exclude singleton. The KIR regions are 19:55200000 to 19:55400000 and GL000209.1, or chr19:55200000–55400000 and chr19_gl000209_random if UCSC (Lee *et al.* 2022) version is used. Additionally, we provide an option to utilize an alt-scaffold-free hg38 reference, enabling users to bypass potential mapping complications associated with alternative contigs. In the hg38 assembly, Graph-KIR automatically targets the KIR region defined by the coordinates chr19:54720000–54870000.

### 2.4 Read mapping

The read mapping parameters we used are similar to HLA typing in HISAT2-genotype hisat2 --no-spliced-alignment --max-altstried 64 --haplotype. In this research, the extracted reads are mapped to the three different graph indexes: “ab2dl1s1,” “ab,” and “split” for the following comparison.

After mapping, we remove reads with multiple mapping, low alignment score (edit distance > 4) or unpaired flag. Those discarded reads mostly came from other KIR genes.

### 2.5 Copy number estimation

The copy number estimator is a composite of multiple distributions. Each distribution represents the read depths of genes with identical copy number. Thus, the distributions correspond with different non-negative integer copy number. Furthermore, the parameters of distributions exhibit a linear relationship with the corresponding copy number. These characteristics rely on the assumption that the reads are uniformly sequenced across the genome and be accurately mapped by the read mapper to their respective genes. For a merged gene pair, e.g. *KIR2DL5A* and *KIR2DL5B*, they are treated as a single gene. In this regard, only one copy number will be reported. The copy number of each gene within the merged pair will be determined after allele typing.

The read depth of a gene is determined as the 75th percentile of the read depths of the positions across the gene. There are alternatives such as average or median. If WES/capture-based samples are provided, Graph-KIR has the option to calculate the depths with the exon part only.

Let $D=\{d_{2\text{DL}1},d_{2\text{DL}2},\ldots ,d_{g}\}$ be the read depth of each gene. Here, we assume the read depths of genes with copy number k follow a normal distribution $P(d|\text{CN}=k)∼N(\mu_{k},\sigma_{k}^{2})$. The parameters of distributions are constrained to $\mu_{k}=k\cdot \mu_{2}/2$ because of the linear relationship mentioned above. Additionally, the standard deviations are adjusted so that read depth is more likely to fit to CN = 2 instead of other copy numbers. Here, the deviations at CN = 2 are kept minimal, while the other deviations are increased to reduce their likelihood. Furthermore, CN = 1 is more likely to occur than CN = 3. To satisfy this preference, we introduce two constants, $C_{n}$ and $C_{p}$, where $C_{n}$ is smaller than $C_{p}$. This allows the equation to take the following form:


(1) $$\sigma_{k}=\sigma_{2}+\sigma_{2}\cdot \{\begin{array}{cc} (2-k)C_{n}, & \text{if}\;k=0,1;\\ (k-2)C_{p}, & \text{otherwise}. \end{array}$$

Once $\mu_{2}$ and $\sigma_{2}$ are set, the distributions are all fixed. The copy number of a gene can be determined by assigning it to the distribution CN = *k* that has the maximum likelihood of observing its read depth:


(2) $${\text{CN}}_{g}=\underset{k}{\text{argmax}}P(d_{g}|\text{CN}=k),\;k=0,1,2,\ldots ,6.$$

The overall likelihood of observing the inferred copy numbers given the distributions can be calculated by multiplying the likelihood of each gene described in the Equation (2). The maximum can be found by changing the variable $\mu_{2}$, the others are set to $\sigma_{2}=0.08$, $C_{p}=0.5$, $C_{n}=0.3$ and read depths are normalized by the maximum one in this study.

Graph-KIR provides an option to assume *KIR3DL3*, one of the framework genes, has two copies and adjust $\mu_{2}$ based on the assumption. Additionally, Graph-KIR offers an additional option to determine the copy number of multiple samples jointly. The only change in this option is that the read depths are calculated from all the samples in the cohort, i.e. $D=\{d_{2\text{DL}1\;\text{sample}1},d_{2\text{DL}2\;\text{sample}1},\ldots ,d_{g\;{\text{sample}}_{n}}\}$.

### 2.6 Allele typing

The allele typing is, indeed, finding a possible set of star alleles according to the variants in the reads. The number of alleles (set size) of each gene is the copy number estimated in the previous step. Graph-KIR determined the set of each gene independently. For indices “ab” and “ab2dl1s1,” the merged gene pair is typed as a unit in this stage.

In the first step of allele typing, Graph-KIR calculates the likelihood of the read originating from a specific allele by matching the variants within the read interval. For any unmatched variant, a probability of 0.001 is assigned, indicating the probability of sequencing errors or mapping errors. The probability is defined as


(3) $$P(\text{allele}|\text{read})=\prod_{v\in {\text{Variants}}_{\text{read}}}\{\begin{array}{cc} 0.999, & \text{if}\;v\in {\text{Variants}}_{\text{allele}}\\ 0.001, & \text{else} \end{array}$$

where ${\text{Variants}}_{\text{allele}}$ is variants inside the allele and ${\text{Variants}}_{\text{read}}$ is variants inside the read. By default, Graph-KIR utilizes all variants across the entire genomic region—including both coding and non-coding sequences—to define the set ${\text{Variants}}_{\text{allele}}$. However, for users focused specifically on 3-digit or 5-digit resolution, an “exon-only” option restricts the analysis to exonic variants only, aligning the calling process with functional allele definitions.

For genes with a copy number equal or >2, Graph-KIR first assesses whether the gene is homozygous by examining all variant positions in the mapped reads for that gene. If no variant position with coverage greater than two-thirds of the sequencing depth has a minor allele frequency (MAF) exceeding 1/2×CN, the gene is classified as homozygous.

For homozygous genes and genes with a single copy number, the gene’s allele type is identified by selecting the allele with the highest likelihood based on the match between the allele and the gene’s reads:


(4) $$\underset{{\;}_{\text{allele}\in {\text{Alleles}}_{g}}}{\text{argmax}}\prod_{\text{read}\in \;\text{reads}}P(\text{allele}|\text{read})$$

where “${\text{Alleles}}_{g}$” is all the alleles of a gene *g*. For a merged gene pair, the symbol “*g*” in “${\text{Alleles}}_{g}$” stands for a gene pair. In other words, the set “${\text{Alleles}}_{g}$” contains all the alleles from both genes in a pair.

For heterozygous genes, the overall probability of the reads being generated from a set of alleles (denoted as “alleles”) is calculating the product of assigning each read to one of the alleles that has the maximum probability:


(5) $$P(\text{alleles}|\text{reads})=\prod_{\text{read}\in \text{reads}}\underset{\text{allele}\in \text{alleles}}{\text{max}}P(\text{allele}|\text{read})$$

The alleles for each gene is then determined by choosing the set that has the highest overall probability from all possible combinations:


(6) $$\underset{{\text{Alleles}}_{{\text{CN}}_{g}}\in \{(a_{1},\ldots ,a_{k})|a_{i}\in {\text{Alleles}}_{g},i=1,2,\ldots ,{\text{CN}}_{g}\}}{\text{argmax}}P({\text{Alleles}}_{{\text{CN}}_{g}}|\text{reads})$$

where “${\text{Alleles}}_{{\text{CN}}_{g}}$” is the determined allele set of a gene. For a merged gene pair, the symbol “*g*” in “${\text{Alleles}}_{g}$” and “${\text{Alleles}}_{{\text{CN}}_{g}}$” stands for a gene pair. After allele typing, the copy numbers of the two genes in a pair are finally determined by counting the number of alleles for each gene.

Instead of examining all combinations which is computationally intensive, our strategy is to iteratively increase alleles set size from one until reaching the estimated copy number. For each iteration, we only preserve the top 600 sets having highest overall probability.

To minimize the error caused by mapping error and sequencing error, Graph-KIR designs an error correction procedure to ignore a variant if it has read depth <3 or the variant is rare (The read depth ratio of alternative over reference type < 0.25).

### 2.7 Simulated data preparation

The same pipeline as PING (Marin *et al.* 2021) is used to generate simulated short reads. In short, two haplotypes are randomly selected from the haplotypes summarized by (Jiang *et al.* 2012) for a human sample. Once the haplotypes are chosen, copy number for each KIR gene can be collected, and then KIR alleles correspond to each gene are randomly selected with replacement. After all the alleles are selected, ART (Huang *et al.* 2012) will generate reads from the sequences of selected KIR alleles. In total, 100 samples are generated and all of them contain about 30× 150 bp paired-end reads with sequencing errors. Note that the sequences generated by this method don’t include the inter-gene sequences because this research mainly focuses on KIR gene content.

### 2.8 HPRC data

The real sample set for evaluating the performance of KIR pipelines are from Human Pangenome Reference Consortium (HPRC) (Wang *et al.* 2022). The CN combinations among the annotated 47 HPRC samples are shown in Fig. S4, available as supplementary data at *Bioinformatics* online. In total, 44 out of 47 samples have WGS short pair-end reads available from NCBI (see Supplement, available as supplementary data at *Bioinformatics* online).

The annotation of HPRC samples used in this research was obtained in a previous study, the SKIRT pipeline (Hung *et al.* 2024). The annotated KIR alleles [the second supplmental table of Hung *et al.* (2024)] were identified by using minimap2 (Li 2018), where allele sequences from the IPD-KIR database were mapped to high-quality phased haplotypes, which were generated by assembling HiFi reads from each HPRC sample. The annotated alleles were further validated by manual examination using IGV (Robinson *et al.* 2011). Notably, numerous novel alleles were found, but for subsequent comparisons, the closest alleles were considered.

### 2.9 Allele comparison

To evaluate the result of allele typing, we compare the predicted alleles with the ground truth alleles at three different resolutions: 7-digit, 5-digit, and 3-digit. A pair of predicted allele and ground truth allele will be marked as correct (True Positive, TP) if they share exactly the same or more digits at the desired level. Additionally, each predicted allele and ground truth allele can only be paired with at most one of its counterpart. Alleles in the predicted set that lack a corresponding match in the ground truth are classified as False Positives (FP), while ground truth alleles missing from the predicted set are classified as False Negatives (FN). Consequently, Recall at each resolution is defined as the number of correctly identified pairs normalized by the total number of ground truth alleles. Precision is defined as the number of correctly identified pairs normalized by the total number of predicted alleles. Finally, the F1-score is calculated as $F_{1}=2\cdot \frac{\text{Precision}\cdot \text{Recall}}{\text{Precision}+\text{Recall}}$.

If a tool outputs multiple sets of alleles when it encounters ambiguity (PING, SakaueKIR), we simply choose the first set to evaluate. It ensures that the number of alleles is equal to its estimated copy number. Another evaluation is to treat all output sets as the called alleles for a sample. This treatment will create lots of False Positive (FP) alleles, but the recall rates will be higher because one of the predicted alleles may match the ground truth allele. In the case of novel alleles, the alleles provided by the tool will be used for comparison.

## 3 Results

### 3.1 Copy number estimation

The performance of five other KIR pipelines and different options of Graph-KIR on copy number estimation was evaluated by 100 simulated data, which has ground truth as it was generated. The difference against the ground truth is calculated by the sum of the absolute difference of each gene across all simulated samples, denoted as “Diff” in Table 1.

**Table 1:** Performance of copy number estimation on 100 simulated samples.^a^^,^^b^

Methods	Total	Diff	Accuracy
Graph-KIR-split	2389	256	0.893
Graph-KIR-ab	2389	42	0.982
Graph-KIR-ab2dl1s1	2389	20	0.992
Graph-KIR-ab2dl1s1-cohort	2389	20	0.992
Graph-KIR-ab2dl1s1-median	2389	26	0.989
Graph-KIR-ab2dl1s1-exon	2389	26	0.989
KIR*KPI	2389	389	0.837
PING-wgs	2389	30	0.987
SakaueKIR	2389	1325	0.445
T1K	2389	321	0.865
Geny	2389	39	0.984

a The intermediate copy number result of PING is obtained from “manualCopyNumberFrame.csv,” which is generated after manually providing the copy number thresholds. KIR*KPI only indicates the presence/absence of a gene but does not provide copy number information. Therefore, we use the predicted haplotypes to infer the copy number of each gene (by “haps.txt”).

b “split” indicates that the graph index contains 17 graphs, where no gene is merged. “ab” indicates that the graph index contains 16 graphs, where *KIR2DL5A* are *KIR2DLB* merged. “ab2dl1s1” indicates that the graph index contains 15 graphs, where *KIR2DL5A* and *KIR2DLB* are merged, and *KIR2DL1* and *KIR2DS1* are merged. “cohort” indicates that copy numbers are jointly called rather than independently from each sample. “median” indicates that the median of read depths is used instead of the 75th percentile. “exon” indicates that only the read depth of the exon part is used. “wgs” indicates the wgs version of PING is used for comparison. Accuracy=1−Diff/Total.

As summarized in Table 1, among the methods of Graph-KIR with three indexes, “ab2dl1s1” (default) has 99.2% accuracy, “ab” has much lower accuracy, and “split” has the lowest accuracy. We conclude that merging similar KIR genes preserved more uniquely mapped reads for read depth calculation, which highly influences the copy number estimation results. Among different configurations, three key points can be concluded:

- Using median value (“-median”) to represent the read depth of a gene is not better than using the 75 percentile (default).
- Determining the copy number by cohort (“-cohort”) and one-by-one version (default) perform equally well.
- Calculating read depth using only the exon region (“-exon”) has an accuracy lower than using full sequence (default).

Among the five methods for comparison, PING (Marin and Hollenbach 2023) has almost perfect copy number prediction when the correct threshold was manually applied. We manually set the threshold by comparing the value against the ground truth of copy numbers. Geny demonstrates similar performance to “Graph-KIR-ab.” The latter uses 16 MSAs from the IPD-KIR database, in which KIR2DL5A and KIR2DL5B have been pre-merged. Other tools like KIR*KPI, SakaueKIR, and T1K all have accuracy below 0.865.

Table 2 presents the accuracy of estimated copy numbers across 44 HPRC samples. The results are similar to Table 1, with Graph-KIR outperforming the other tools. The superior performance of Graph-KIR’s per-sample estimation, compared to cohort estimation, can be attributed to the varying sequence depths across some of the HPRC samples (Table S1, available as supplementary data at *Bioinformatics* online), i.e. not always 30×.

**Table 2:** Performance of copy number estimation on 44 HPRC samples.^a^

Methods	Total	Diff	Accuracy
Graph-KIR-ab2dl1s1	832	34	0.959
Graph-KIR-ab2dl1s1-cohort	832	45	0.945
PING-wgs	832	106	0.872
Geny	832	88	0.894

a “ab2dl1s1” indicates that the graph index contains 15 graphs, where *KIR2DL5A* and *KIR2DLB* are merged, and *KIR2DL1* and *KIR2DS1* are merged. “cohort” indicates that copy numbers are jointly called rather than independently from each sample. “wgs” indicates the wgs version of PING is used for comparison. Accuracy=1−Diff/Total.

### 3.2 Allele typing

The performance of allele typing using Graph-KIR with different configurations and other KIR pipelines across the 100 simulated samples are shown in Table 3. The results are presented at 3 resolutions: 7-digit, 5-digit, and 3-digit level. Here, Graph-KIR utilizes the “ab2dl1s1” index as its default configuration.

**Table 3:** Performance evaluation of different KIR allele typing methods on 100 simulated samples across 3-digit, 5-digit, and 7-digit resolutions.^a^

Methods	Recall	Precision	F1
**3-digit (alleles = 2389)**			
GraphKIR	**0.9711**	0.9711	0.9711
Ping-wgs-ans	0.9393	0.9565	0.9478
SakaueKIR	0.4475	0.4913	0.4684
SakaueKIR-all	0.5722	0.0256	0.0490
SakaueKIR-ans	0.5119	0.5646	0.5370
SakaueKIR-ans-all	0.5659	0.0511	0.0937
T1K	0.8585	0.9785	0.9146
Geny	0.9686	**0.9805**	**0.9745**
**5-digit (alleles = 1946)**			
GraphKIR	**0.9697**	**0.9777**	**0.9737**
Ping-wgs-ans	0.9193	0.9362	0.9277
SakaueKIR	0.4445	0.4663	0.4551
SakaueKIR-all	0.5704	0.0410	0.0765
SakaueKIR-ans	0.5092	0.5380	0.5232
SakaueKIR-ans-all	0.5586	0.0681	0.1214
T1K	0.8458	0.9717	0.9044
Geny	0.9594	0.9684	0.9639
**7-digit (alleles = 1752)**			
GraphKIR	**0.9121**	**0.9237**	**0.9179**
Ping-wgs-ans	NA	NA	NA
SakaueKIR	0.0611	0.0657	0.0633
SakaueKIR-all	0.3014	0.0394	0.0697
SakaueKIR-ans	0.0685	0.0759	0.0720
SakaueKIR-ans-all	0.2928	0.0450	0.0780
T1K	0.2380	0.2762	0.2557
Geny	0.6667	0.6655	0.6661

a “all” indicates all allele sets in output of a tool are treated as called alleles; otherwise, only the first set is used. “ans” indicates the copy number is assigned by peeking the ground truth. “wgs” indicates the wgs version of PING is used for comparison. Graph-KIR and T1K are both based on IPD-KIR version 2.10.0. PING-wgs is based on IPD-KIR 2.11.0, and Geny is 2.12.0. SakaueKIR is based on 2.7.0. NA: not available. Bold values indicate the highest score for each evaluation metric within each resolution level.

Graph-KIR consistently demonstrates high accuracy across multiple resolutions, achieving F1-scores of 0.9737 and 0.9179 at the 5-digit and 7-digit levels, respectively. In comparison, Geny achieves the highest F1-score at 3-digit resolution (0.9745) and performs well at 5-digit resolution (0.9639), but its accuracy drops significantly to 0.6661 at the 7-digit level. Other tools show further limitations: PING achieves a 5-digit F1-score of 0.9277 but cannot provide 7-digit resolution, while T1K maintains a 5-digit F1-score of 0.9044 but exhibits very low accuracy (0.2557) at the 7-digit level. The false negative of T1K is quite large because it predicts at most 2 alleles per gene which is not always true for KIR genes. SakaueKIR has the lowest F1-score (0.5232) at 5-digit resolution, mainly because most of the alleles in simulated data are novel for the IPD version 2.7.0. The limitation of some tools is there’s no command for updating its database.

Table 4 shows the allele typing results compared to the annotated HPRC samples. Only PING, Geny, and Graph-KIR are selected for comparison because of their superior performance on the simulated data. Across the 44 HPRC samples, Graph-KIR demonstrates better accuracy at 7-digit resolution, while Geny performs better at the 3-digit and 5-digit levels. A significant challenge in using the HPRC dataset for benchmarking is the prevalence of fusion genes and novel alleles not yet documented in IPD-KIR version 2.12.0. In these complex cases, Graph-KIR achieves a higher 3-digit F1-score (0.8209) when the “exon-only” typing mode is utilized.

**Table 4:** Performance evaluation of different KIR allele typing methods on HPRC data across different resolutions.^a^

Methods	Recall	Precision	F1-score
**3-digit (alleles = 832)**			
GraphKIR-exon-hg19	**0.8209**	0.8209	0.8209
GraphKIR-exon-hg38	0.8197	0.8197	0.8197
GraphKIR-hg19	0.7596	0.7596	0.7596
GraphKIR-hg38	0.7584	0.7584	0.7584
PING-wgs-ans	0.6526	**0.8902**	0.7531
Geny	0.8005	0.8856	**0.8409**
**5-digit (alleles = 720)**			
GraphKIR-exon-hg19	0.7778	0.8396	0.8075
GraphKIR-exon-hg38	0.7778	0.8396	0.8075
GraphKIR-hg19	0.7583	0.7994	0.7783
GraphKIR-hg38	0.7597	0.8009	0.7798
PING-wgs-ans	0.6458	0.8579	0.7369
Geny	**0.8153**	**0.8696**	**0.8416**
**7-digit (alleles = 248)**			
GraphKIR-hg19	0.7782	0.6108	0.6844
GraphKIR-hg38	**0.7823**	**0.6120**	**0.6867**
PING-wgs-ans	NA	NA	NA
Geny	0.6048	0.5282	0.5639

a “exon” indicates Graph-KIR adopts only exonic variants when performing allele typing (the “exon-only” mode). “hg19” and “hg38” indicates the genome versions used. “ans” indicates the copy number is assigned by peeking the ground truth. “wgs” indicates the wgs version of PING is used for comparison. NA: not available. Bold values indicate the highest score for each evaluation metric within each resolution level.

Beyond evaluating overall metrics, we sought to explore the potential underlying reasons for the substantial performance gap observed between simulated and real-world datasets at 7-digit resolution. To achieve this, we conducted a systematic ablation analysis by incrementally removing complex genomic cases to isolate the impact of specific biological features on calling accuracy. Specifically, we examined how gene fusions, nonsynonymous variants in the CDS, segmental deletions, and synonymous variants influenced the F1-scores. We also evaluated the challenges posed by non-CDS variants and alleles that only match IPD-KIR CDS-only records.

As shown in Table S2, available as supplementary data at *Bioinformatics* online, when these complex structural variations and novel polymorphisms are removed from the HPRC assemblies, the performance of both Graph-KIR (F1-score from 0.6867 to 0.8140) and Geny (F1-score from 0.5639 to 0.6737) improves significantly. This diagnostic approach confirms that both methods are similarly susceptible to specific high-complexity features—most notably gene fusion events, large segmental duplications, and the presence of alleles not yet documented in the standard IPD-KIR database.

Additionally, we investigated the impact of sequencing depth on allele-calling performance by categorizing the 44 HPRC samples into three groups based on the read depths detailed in Table S1, available as supplementary data at *Bioinformatics* online. The resulting performance metrics across varying resolutions are presented in Table S3, available as supplementary data at *Bioinformatics* online. Our analysis indicates that lower sequencing depths (<30×) do not significantly compromise performance. Conversely, the highest-depth group (>40×) exhibited slightly reduced accuracy; this group includes some early-released HPRC samples, which may possess lower overall sequence quality that adversely affects calling precision.

To complement this stratification with a controlled assessment of low coverage, we downsampled 36 HPRC samples (native depth > 30×) to 5×, 10×, 15×, 20×, and 30× for allele typing (Table S4, available as supplementary data at *Bioinformatics* online and Fig. S5, available as supplementary data at *Bioinformatics* online). The F1-score rises steadily with depth at all three resolutions: 5× is markedly degraded, results become usable from 10× onward, and 15× provides consistent performance across all resolutions, with performance continuing to improve up to the full native depth. These results characterize the low-coverage behavior of Graph-KIR and support its use on large-scale short-read WGS datasets that may include lower-coverage samples, identifying ∼10× as the lowest usable depth and ∼15× as a practical lower bound for consistent typing across resolutions.

## 4 Discussion

### 4.1 Similarity between KIR genes

Genes with high similarity often lead to reads being mapped to multiple positions, introducing ambiguity and complicating subsequent analyses. We attempted to address this challenge by merging similar genes before building graph index for read mapping, such as *KIR2DL5A* and *KIR2DL5B*, resulting in improved performance in both steps.

For Graph-KIR, merging *KIR2DL1* and *KIR2DS1* is critical, especially for copy number estimation. Many reads are mapped to both *KIR2DS1* and *KIR2DL1* in the “ab” index. Only half (51%) of the reads mapped to *KIR2DS1* and 87% of the reads mapped to *KIR2DL1* are uniquely mapped. Removing the multi-mapped reads significantly influenced the read depth of these genes, leading to errors in copy number estimation. In contrast, in the “ab2dl1s1” index, 87% of *KIR2DL1* reads and 84% of *KIR2DS1* reads are retained.

The analysis above is based on simulated data. To verify it on real sequencing data and extend it from the *KIR2DL1*/*KIR2DS1* pair to all genes, we report the per-gene unique-mapping rate under the merged (ab2dl1s1, 15 MSAs), partially split (ab, 16 MSAs), and fully split (split, 17 MSAs) indices, together with per-gene copy-number accuracy and allele-typing F1-score, in Table S5, available as supplementary data at *Bioinformatics* online. Splitting the index collapses the unique-mapping rate only for the high-homology genes while leaving all other genes essentially unchanged, and the joint copy-number accuracy of both merged pairs is perfect under the recommended index. Within this index, unique-mapping rate is uncorrelated with allele-typing F1, indicating that once merging preserves read depth, the remaining limits on accuracy arise from allele-level sequence similarity rather than from read mapping.

### 4.2 Limitation

Graph-KIR shows lower performance on real data compared to simulated data, primarily due to the presence of novel alleles. Removing these novel alleles from the comparison set improves accuracy; however, even after filtering out all novel alleles, as shown in the final section of Table S2, available as supplementary data at *Bioinformatics* online, Graph-KIR’s allele typing accuracy remains below the levels achieved on simulated data.

After examining the read mapping results in IGV, we found that despite the use of graph mapping, long deletions remain challenging to be distinguished. For example, in KIR3DP1*0030202, which has several long deletions near the 5’ end of the graph backbone, almost no reads were mapped to the first 2000 base pairs, resulting in missing information such as the deletion lengths and the sequences between deletions. This made it difficult to distinguish KIR3DP1*0030202 from alleles with identical sequences beyond the first 2000 base pairs. This limitation accounts for about 5% decrease in the 7-digit allele typing of Recall in Table S2, available as supplementary data at *Bioinformatics* online. In other words, we might have the chance to increase Recall by 5% if we can enhance the mapping results in this region.

While multi-mapped reads are currently discarded after the Graph-KIR read mapping steps to enhance precision, distinguishing them from multiple mapping positions could lead to improved performance in both precision and recall rate.

While Graph-KIR optimizes short-read analysis, Illumina sequencing remains inherently limited in resolving highly paralogous genes and complex structural haplotypes compared to long-read technologies. Although long-reads excel at full-range phasing and resolving identical repeats, Graph-KIR provides a robust and cost-effective solution for high-resolution KIR typing in the large-scale short-read WGS datasets that dominate current clinical and population studies.

### 4.3 Evaluation of speed

Graph-KIR uses existing tools for computation-intensive tasks and only customizes the core function of copy number estimation and allele typing. For example, BWA-MEM for read mapping and extraction, HISAT2 for graph mapping and Samtools for read depth calculation. These optimized tools speed up the execution time of Graph-KIR. In the test of 100 simulated samples, it takes Graph-KIR 41 min to sequentially process each sample, and takes 8 min if run in parallel e.g. GNU Parallel (Tange 2011) because Graph-KIR can predict KIR allele per sample independently. In contrast, it takes PING about 52 h 20 min for the 100 samples, including the time for the in-house program to automatically provide the thresholding information. All the experiments were conducted on high-performance computer TAIWANIA3’s SLURM partition ngs92G, which has 14 virtual cores and 92G virtual memory.

## 5 Conclusion

Graph-KIR is a brand new pipeline for typing KIR alleles at 7-digit resolution from short-read WGS data. With a proper index and configurations selected, the polymorphism of KIR genes can be effectively handled by the tool optimized for graph mapping. The procedure to determine copy numbers can be done with only one sample, this feature enables KIR research without collecting lots of samples. When evaluated with both simulated and real data, Graph-KIR demonstrated better performance over other existing KIR tools in precise gene copy number estimation and full-resolution (7-digit) allele calling. The proposed pipeline can be modified and adapted in the near future for HLA allele typing, especially for genes have copy number issue like *DRB3*, *DRB4* and *DRB5*.

## Supplementary Material

btag521_Supplementary_Data

## Data Availability

The simulated data used in this study can be generated using the code kg_create_data.py, located in the research folder of the Graph-KIR repository. The real data used in this study are HPRC data (Wang *et al.* 2022). Graph-KIR and the related research codes are publicly available at https://github.com/linnil1/KIR_graph, and an archived snapshot of the code and benchmarking results used in this study is deposited at Zenodo (DOI: https://doi.org/10.5281/zenodo.20759143).
