# Supplementary material for: Graph-KIR: graph-based KIR copy number estimation and allele calling using short-read sequencing data
Source: Bioinformatics. 2026 Jul 21;42(7):btag521. doi: 10.1093/bioinformatics/btag521 (PMC13423236; doi:10.1093/bioinformatics/btag521)
Supplement: btag521_Supplementary_Data [file btag521_supplementary_data.pdf]

Supplementary Material

Graph-KIR: graph-based KIR copy number estimation and allele calling using short-read sequencing data

* MSA

Similar alleles are defined as those that share at least the first 5 digits. If no such alleles are found, those sharing the first 3 digits are considered instead. If no similar alleles with at least the first 3 digits are identified, the consensus is calculated using all full-length alleles within the same gene.

If it is required to merge two or more MSAs, Graph-KIR will split the MSA into blocks of MSAs by introns and exons. The MSA blocks correspond to the same intron or exon regions are realigned separately by Muscle (Edgar 2004) with default parameters. Then, the merged MSA is constructed by concatenating the realigned blocks in order. Splitting the MSA, such as separating *KIR2DL5A* and *KIR2DL5B* from *KIR2DL5*, is a straightforward process. The split MSAs only include alleles with the corresponding gene name, and any column only contains gap is removed.

Once MSAs are prepared, the consensus can be calculated by selecting the base with maximum frequency in each column while ignoring gaps. These consensus sequences are then saved in the .fasta format as the graph backbone. The variants, called from each star allele against the backbone, are saved in .snp and .haplotype files. Note that variants with frequency greater than 0.1 will be reserved in the graph index. Graph indexes are then built by running 'hisat-build' with the backbone and variants files mentioned above. The MSA operations previously described are from another open-source project developed by the same authors of Graph-KIR. The codes are available at `https://github.com/linnil1/pyHLAMSA`.

* Read mapping

The effectiveness of Graph-KIR heavily relies on the precision of graph read mapping. Improvements in read mapping can subsequently enhance the results of both copy number estimation and allele typing. To assess the effectiveness of read mapping, especially when multiple mapping for a read is possible, we utilize metrics including False Discovery Rate (FDR), recall, precision, and alignment rate. FDR represents the proportion of incorrect mapping positions of all the reads including multiple mapping. Recall is the rate of reads that have at least one correct mapping position. Precision is 1 - FDR. The alignment rate quantifies the proportion of mapped reads among all the reads of each gene.

Various methods were assessed here, including linear mapping (BWA-MEM, Bowtie 2) and graph mapping (Graph-KIR) with different indexes ('split', 'ab', 'ab2dl1s1'). This evaluation is conducted on the 100 simulated samples. All setups with the 'unique' option (points in cross) have a smaller recall but also a smaller FDR compared to the 'all' option (points in circle) shown in Fig. S2. This phenomenon indicates that filtering multiple mapped reads leads to a decreased numerator in recall, but it simultaneously decreases the number of false positives, resulting in smaller numerator and denominator in FDR. Overall, recall rates slightly dropped while FDRs significantly decreased.

In Fig. S2, Bowtie 2 has the highest false discovery rate (FDR = 0.81). Among the three different graph indexes, Graph-KIR based on 'split' (17 graphs) has the highest FDR (FDR = 0.20). Index 'ab' (16 graphs) merges two similar genes *KIR2DL5A* and *KIR2DLB* to reduce FDR to 0.12. Index 'ab2dl1s1' further lowers the FDR to 0.09 by merging *KIR2DL1* and *KIR2DS1*. These three results show that merging similar genes to eliminate sequence redundancy and allele similarity between indexes can decrease the FDR of the graph mapper. The index 'ab2dl1s1' of Graph-KIR is a little less accurate than BWA-MEM ('BWA-MEM', FDR=0.07). However, the unique-filtering version 'ab2dl1s1, unique' decreases FDR (0.09 to 0.008) at the expense of a lower recall rate (0.97 to 0.89). Applying the same filtering on BWA-MEM ('BWA-MEM, unique') does not result in an apparent decrease in the FDR (0.071 to 0.069).

To compare the read mapping results of different strategies thoroughly, we also take precision and alignment rate at gene-level into account, as shown in Fig. S3. In this figure, all methods have been filtered using the 'unique' option. Each method has 17 points, representing 17 KIR genes. The results demonstrate that the proposed method, Graph-KIR with the 'ab2dl1s1' index, has significantly higher precision than other methods. Although BWA-MEM exhibits higher alignment rate, which means preserving more reads, some of the retained reads may include noise at downstream allele typing due to the lower precision. In conclusion, Graph-KIR outperforms other linear mapping methods in terms of precision, FDR, and recall rate. Among all the indexes evaluated, 'ab2dl1s1' stands out as the most suitable one.

* Sample IDs of the real data

In total, 44 out of 47 samples have WGS short pair-end reads available from NCBI. Here is the list of HPRC samples: HG002, HG00438, HG005, HG00621, HG00673, HG00733, HG00735, HG00741, HG01071, HG01106, HG01109, HG01175, HG01243, HG01258, HG01358, HG01361, HG01891, HG01928, HG01952, HG01978, HG02055, HG02080, HG02109, HG02145, HG02148, HG02257, HG02572, HG02622, HG02630, HG02717, HG02723, HG02818, HG02886, HG03098, HG03453, HG03486, HG03492, HG03516, HG03540, HG03579, NA18906, NA19240, NA20129, NA21309. The average sequencing depth of each sample is shown in Table S1. Analysis was restricted to the genomic coordinates chr19:54,720,000–54,860,000 (GRCh38), with depth metrics derived using the samtools coverage utility.

References

Edgar RC. MUSCLE: a multiple sequence alignment method with reduced time and space complexity. *BMC Bioinformatics* 2004;**5**:1–19.

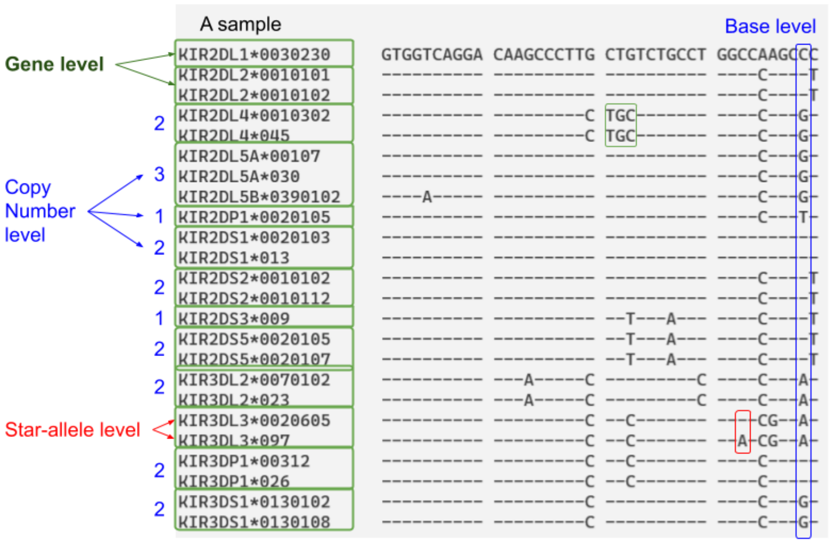

Fig. S1. Demonstration of KIR complexity at four levels using one of the simulated samples. These levels comprise the base level, gene level, star-allele level, and copy number level, with the copy number level highlighting the count of star alleles within a single KIR gene. The symbol '-' stands for a position identical to the base of *KIR2DL1**0030230.

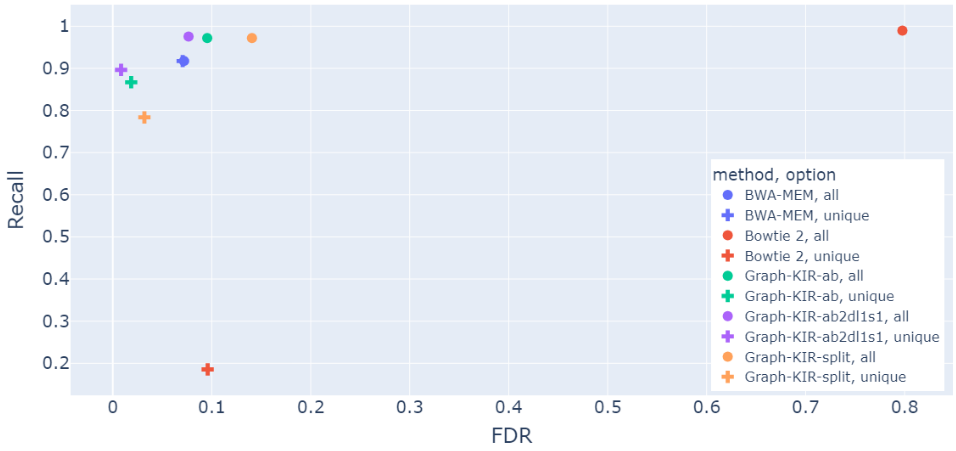

Fig. S2. The effectiveness of various mapping strategies is assessed based on False Discovery Rate (FDR) and Recall of the reads across 100 simulated samples. Recall is the ratio of the reads having at least one correct mapping position divided by the total number of reads. Conversely, FDR is the percentage of mapping positions that are incorrect. For the methods with the 'unique' suffix, multi-mapped reads are disregarded, while 'all' indicates all the reads are considered. Both BWA-MEM and Bowtie 2 use 'ab2dl1s1's backbone as the index.

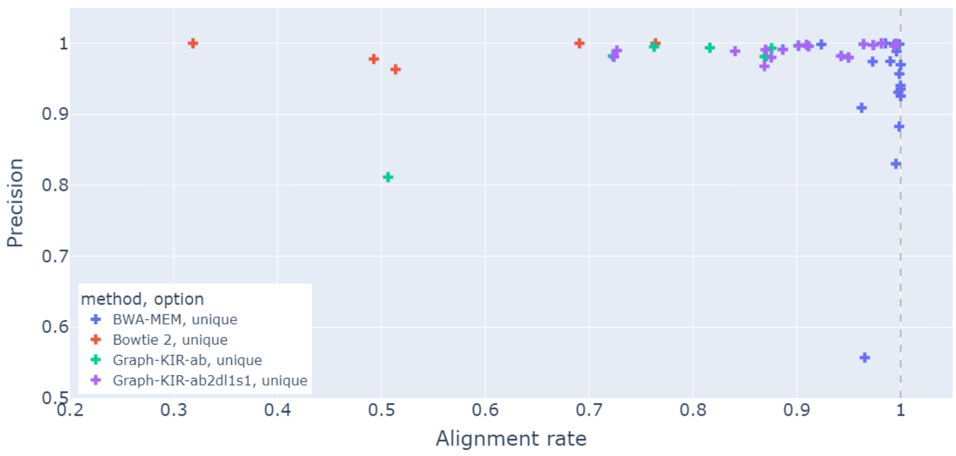

Fig. S3. Precision is plotted against alignment rate for each method at gene level, with 17 points per method. Precision is the proportion of reads that are correctly mapped to their originating locations. The alignment rate quantifies the proportion of mapped reads among all the reads of each gene. For the methods with the 'unique' suffix, multi-mapped reads are disregarded. Both BWA-MEM and Bowtie 2 use 'ab2dl1s1's backbone as the index.

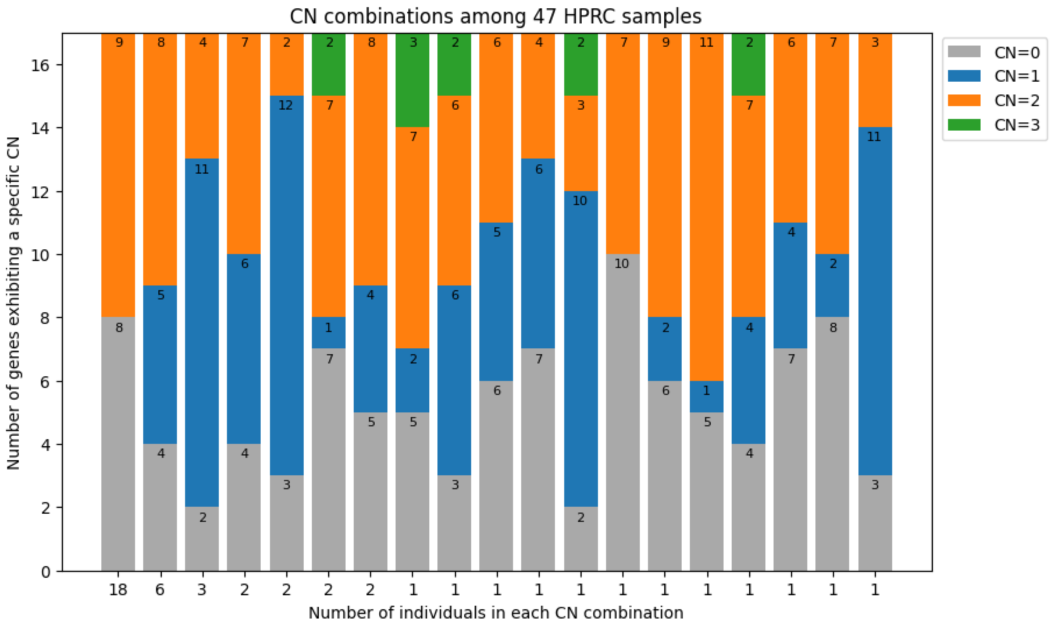

Fig. S4. The CN combinations among the annotated 47 HPRC samples.

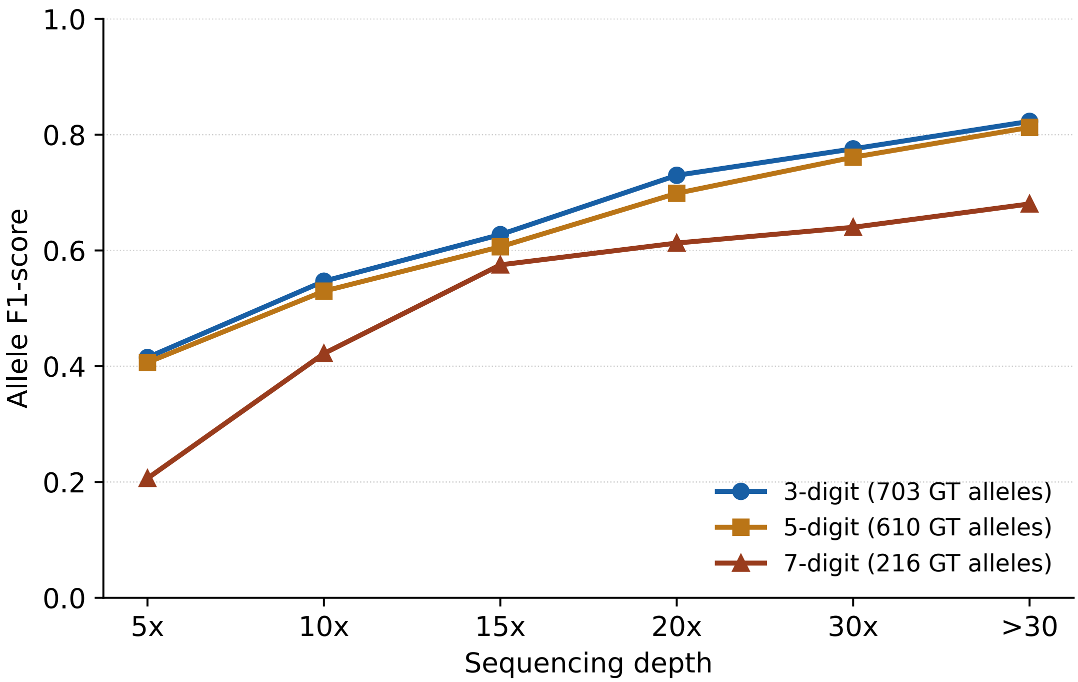

Fig. S5. Allele-typing F1-score of Graph-KIR as a function of sequencing depth, from a downsampling experiment on 36 HPRC samples with native depth >30x (mean 36.7x, range 31.2–70.5x); ">30" denotes the full native depth, and its higher mean depth accounts for the further improvement over the 30x level. F1 increases steadily with depth at all three resolutions. The number of evaluable ground-truth alleles at each resolution is shown in parentheses in the legend. These 36 samples comprise all HPRC samples with native depth above 30x in this study (Supplementary Table S1).

Table S1. Average sequencing depth (Arg. Depth) for each HPRC sample used in the study.

Sample	Avg. Depth	Sample	Avg. Depth
HG002	45.22	HG02109	61.10
HG005	36.67	HG02145	34.77
HG00438	33.09	HG02148	31.23
HG00621	32.04	HG02257	36.38
HG00673	31.64	HG02572	34.83
HG00733	40.75	HG02622	41.60
HG00735	29.27	HG02630	44.60
HG00741	24.99	HG02717	41.20
HG01071	33.51	HG02723	29.53
HG01106	34.25	HG02818	36.93
HG01109	34.12	HG02886	37.36
HG01175	28.92	HG03098	26.47
HG01243	17.40	HG03453	31.84
HG01258	32.60	HG03486	33.44
HG01358	25.68	HG03492	33.11
HG01361	33.31	HG03516	32.75
HG01891	33.24	HG03540	32.65
HG01928	36.09	HG03579	32.17
HG01952	34.16	NA18906	31.63
HG01978	35.09	NA19240	28.16
HG02055	32.41	NA20129	31.15
HG02080	35.33	NA21309	70.48

Table S2. Performance evaluation at 7-digit resolution under varying filter conditions (HPRC dataset). Successive filters were applied to remove complex variations; the specific types of excluded alleles are listed in the 'Filter' column. Values in parentheses represent the total count of alleles retained for evaluation after each filtering step.

Filter	Methods	Recall	Precision	F1-score
**-fusion** (alleles=248)				
	GraphKIR-hg19	0.7782	0.6146	0.6868
	GraphKIR-hg38	**0.7823**	**0.6159**	**0.6892**
	PING-wgs-ans	NA	NA	NA
	Geny	0.6048	0.5319	0.5660
**-fusion -#** (alleles=237)				
	GraphKIR-hg19	0.7890	0.6516	0.7137
	GraphKIR-hg38	**0.7932**	**0.6528**	**0.7162**
	PING-wgs-ans	NA	NA	NA
	Geny	0.6160	0.5573	0.5852
**-fusion -# -+ -=** (alleles=222)				
	GraphKIR-hg19	0.8018	0.7841	0.7929
	GraphKIR-hg38	**0.8063**	**0.7851**	**0.7956**
	PING-wgs-ans	NA	NA	NA
	Geny	0.6081	0.6308	0.6193
**-fusion -# -+ -= -$** (alleles=146)				
	GraphKIR-hg19	**0.7945**	**0.8345**	**0.8140**
	GraphKIR-hg38	**0.7945**	**0.8345**	**0.8140**
	PING-wgs-ans	NA	NA	NA
	Geny	0.6575	0.6906	0.6737

fusion: a gene having fusion with another gene #: an allele having nonsynonymous variant(s) in CDS, segmental deletion or fusion with another gene +: a genomic allele matching an IPD-KIR CDS-only allele =: an allele having synonymous variant(s) in CDS $: an allele having variant(s) in non-CDS NA: not available

Table S3. Performance comparison of Graph-KIR and Geny across different sequencing depths and resolutions. For Graph-KIR, the 'exon-only' mode is adopted for 3-digit and 5-digit allele calling.

Resolution	Depth	Samples	Alleles	Graph-KIR			Geny		
				**Recall**	**Precision**	**F1-score**	**Recall**	**Precision**	**F1-score**
3-digit	>40x	7	153	0.7895	0.7843	0.7869	0.5724	0.8056	0.6693
	30-40x	29	550	0.8364	0.8348	0.8356	0.8527	0.9002	0.8758
	<30x	8	113	0.8288	0.8214	0.8251	0.8559	0.8879	0.8716
5-digit	>40x	7	122	0.7686	0.8304	0.7983	0.6364	0.8462	0.7265
	30-40x	29	488	0.7869	0.8496	0.8170	0.8422	0.8708	0.8563
	<30x	8	94	0.7826	0.8000	0.7912	0.9130	0.8660	0.8889
7-digit	>40x	7	37	0.7027	0.4643	0.5591	0.3784	0.4000	0.3889
	30-40x	29	179	0.7709	0.6479	0.7041	0.6201	0.5415	0.5781
	<30x	8	28	0.8929	0.5952	0.7143	0.7857	0.5500	0.6471

Table S4. Allele-typing F1-score of Graph-KIR across downsampled depths (36 HPRC samples, native depth >30x). ">30" denotes the full native depth.

Depth	3-digit	5-digit	7-digit
5x	0.415	0.407	0.206
10x	0.547	0.530	0.422
15x	0.627	0.606	0.575
20x	0.730	0.699	0.613
30x	0.776	0.761	0.640
>30	0.823	0.813	0.680

Table S5. Per-gene unique-mapping rate, copy-number (CN) accuracy, and allele-typing F1-score for Graph-KIR on HPRC real-data samples ($n = 44$). The unique-mapping rate is defined as the fraction of mapped reads that are not multi-mapped to different positions. It is reported under three indices with progressively fewer merged gene pairs: `ab2dl1s1` (15 MSAs; *KIR2DL1*/*KIR2DS1* and *KIR2DL5A*/*KIR2DL5B* merged), `ab` (16 MSAs; only *KIR2DL5A*/*KIR2DL5B* merged), and `split` (17 MSAs; no merging). As the index is split, the unique-mapping rate collapses only for the high-homology genes (*KIR2DS1*: 88.5% → 38.4% when separated from *KIR2DL1*; *KIR2DL5A*/*KIR2DL5B*: 96.0% → 13.3%/41.2% when separated), while all other genes remain essentially unchanged. CN accuracy and F1-score are computed under the recommended 15-MSA index. For a merged pair, copy number cannot be assigned to the two constituent genes separately and is evaluated jointly; the joint CN accuracy is perfect (1.000) for both pairs and is reported for both constituent genes (and likewise the merged unique-mapping rate). Within the recommended index, unique-mapping rate is uncorrelated with allele-typing F1 (Pearson $r \approx 0$); for example, *KIR2DL2* has the lowest unique-mapping rate (62.4%) yet attains perfect F1 at 5- and 7-digit resolution, whereas *KIR3DL3* has a high unique-mapping rate (99.7%) but a low 7-digit F1 (0.485). † marks genes belonging to a merged pair. "—" indicates no alleles are defined at 7-digit resolution, so no F1 can be computed.

	Unique-mapping rate (%)			CN	Allele F1-score		
Gene	15-MSA	16-MSA	17-MSA	acc.	3-digit	5-digit	7-digit
KIR2DL1†	88.5	84.6	84.5	1.000	0.846	0.806	0.755
KIR2DL2	62.4	63.7	63.6	0.950	0.974	1.000	1.000
KIR2DL3	91.1	90.7	90.8	1.000	0.910	0.916	—
KIR2DL4	99.9	99.9	99.9	1.000	0.943	0.963	0.792
KIR2DL5A†	96.0	96.0	13.3	1.000	0.588	0.588	0.667
KIR2DL5B†	96.0	96.0	41.2	1.000	0.667	0.720	0.222
KIR2DP1	99.4	99.4	99.4	0.987	0.876	0.907	0.588
KIR2DS1†	88.5	38.4	38.3	1.000	0.514	0.692	0.182
KIR2DS2	79.6	80.9	81.0	1.000	0.944	0.897	0.600
KIR2DS3	73.8	73.6	73.5	1.000	1.000	0.875	—
KIR2DS4	92.3	92.2	92.2	1.000	0.945	0.889	0.800
KIR2DS5	80.8	80.9	81.0	1.000	0.588	0.571	0.200
KIR3DL1	95.0	95.0	95.0	1.000	0.931	0.928	0.784
KIR3DL2	99.4	99.4	99.4	0.977	0.782	0.714	0.627
KIR3DL3	99.7	99.7	99.7	1.000	0.693	0.611	0.485
KIR3DP1	97.7	97.7	97.7	1.000	0.575	0.529	0.705
KIR3DS1	86.0	86.0	85.5	1.000	1.000	1.000	0.750
